# The Impact of Polycystic Ovary Syndrome on Gestational Diabetes Mellitus, Disease Knowledge, and Health Behaviors

**DOI:** 10.3390/healthcare13070717

**Published:** 2025-03-24

**Authors:** Hye Jin Kim, Eui Hyeok Kim, Eungil Ko, Sojung Park, Yaelim Lee

**Affiliations:** 1 Department of Administration, Ilsan CHA Hospital, Goyang-si 10414, Gyeonggi-do, Republic of Korea; cometkimhj@gmail.com; 2 Department of Obstetrics and Gynecology, Ilsan CHA Hospital, Goyang-si 10414, Gyeonggi-do, Republic of Korea; raksumi10@gmail.com; 3 Department of Nursing, Asan Medical Center, Seoul 05505, Republic of Korea; dmsrlf3141@catholic.ac.kr; 4 College of Nursing, The Catholic University of Korea, Seoul 06591, Republic of Korea; sojung1226@gmail.com

**Keywords:** polycystic ovary syndrome, diabetes mellitus, metabolic syndrome, health knowledge, attitudes, practice, risk factors

## Abstract

Background/Objectives: Polycystic ovary syndrome (PCOS) is a common endocrine disorder that increases the risk of gestational diabetes mellitus (GDM). This study aims to assess the correlation between PCOS and GDM and to identify associated healthcare needs. Methods: A mixed-methods approach was used. The retrospective study analyzed 2635 medical records of women aged 19–45 who underwent fertility treatments (2020–2023). The prospective study (2023–2024) assessed PCOS and GDM knowledge, nutrition, and physical activity among three groups: PCOS, GDM, and normal pregnancies. Results: Women with PCOS had a significantly higher BMI (*p* < 0.001) and an increased risk of GDM (28.1% vs. 10.6%, *p* < 0.001), with a 2.94-fold higher likelihood (95% CI: 2.22–3.90). Preterm birth (*p* = 0.029) and multiple pregnancies (*p* = 0.014) were also more common. The GDM group demonstrated better nutritional habits (*p* = 0.017), while the PCOS group showed higher physical activity levels (*p* < 0.001). Greater disease knowledge correlated with healthier behaviors. Conclusions: PCOS is a strong risk factor for GDM and adverse pregnancy outcomes. Targeted education and lifestyle interventions are crucial for improving maternal and neonatal health. Future research should focus on long-term metabolic management in women with PCOS.

## 1. Introduction

Infertility is defined as the inability to conceive after one year of regular, unprotected intercourse [1], with a reported global prevalence ranging from 1.9% to 21.1% [2]. In South Korea, 153,085 cases of female infertility were reported in 2022, with a 17.5% increase in women seeking fertility treatment from 2018 to 2022, as per a 2023 report [3]. Furthermore, Korea’s fertility rate has persistently declined, falling into an “ultra-low fertility rate” category (below 1.3) in 2002 and reaching a record low of 0.78 in 2022 [4]. This trend has heightened social concern around infertility and childbirth, prompting new support policies for fertility treatment [5].

Disease-related infertility in women is often linked to ovulatory disorders, endometriosis, and pelvic diseases, including uterine anomalies [6]. Polycystic ovary syndrome (PCOS) is a major cause of ovulatory infertility and affects 5–10% of women of reproductive age, making it the most common endocrine disorder in this population [7]. In 2022, PCOS accounted for 63,701 cases of infertility in South Korea, the largest share among infertility-related conditions [3]. PCOS is a highly heterogeneous disorder with multiple phenotypic variations, influenced by genetic, environmental, and hormonal factors [8]. PCOS is often associated with chronic low-grade inflammation and metabolic disruptions [9]. Its causes are not fully understood, though lifestyle and genetic factors play key roles [9]. PCOS is typically marked by symptoms such as hirsutism, acne, obesity, menstrual irregularities, and infertility [10,11].

PCOS patients also have a high prevalence of gestational diabetes mellitus (GDM), which, along with PCOS, is linked to adverse birth outcomes such as preterm birth and preeclampsia [12,13]. GDM is defined as glucose intolerance first identified during pregnancy, typically occurring in the second or third trimester. It is associated with an increased risk of maternal and neonatal complications, including fetal overgrowth, cesarean delivery, and a higher risk of developing type 2 diabetes postpartum [14]. Research shows that PCOS patients undergoing frozen embryo transplantation have a higher risk of GDM and neonatal complications, underscoring the need for added pregnancy care [13]. Despite the impact of PCOS, there are limited data on pregnancy outcomes and GDM risk factors in PCOS patients. However, population-based studies indicate that the prevalence of GDM has been increasing worldwide, with rates varying depending on screening criteria and population characteristics [15]. Furthermore, while GDM is more common in women with PCOS due to underlying insulin resistance, it also affects a significant proportion of women without PCOS, highlighting the need for universal screening and targeted interventions [16].

Effectively managing weight and insulin resistance is crucial for patients with PCOS, as combining regular high-intensity exercise with balanced nutrition significantly impacts treatment outcomes [17]. Nutritional education is essential for GDM patients, and exercise-based management may reduce postpartum type 2 diabetes risks [18]. Several prospective and retrospective studies suggest that acquiring knowledge about nutrition, exercise, and the disease itself contributes to lifestyle changes and disease awareness, which in turn impact health outcomes associated with the management of PCOS and GDM.

Our hypotheses were as follows: (1) There would be differences in gestational diabetes prevalence and risk factors between the PCOS group and the non-PCOS group. (2) Demographic and pregnancy-related characteristics, as well as knowledge of PCOS and GDM, would influence nutritional status. (3) Demographic and pregnancy-related characteristics, along with knowledge of PCOS and GDM, would affect physical activity.

## 2. Materials and Methods

### 2.1. Study Design

This study utilized both a retrospective and a prospective cohort study design. For the retrospective cohort study, data were collected from the electronic medical records of patients diagnosed with PCOS who had undergone post-treatment pregnancies and deliveries at the A Hospital fertility center between 1 January 2020, and 31 December 2023.

**Figure 1:**
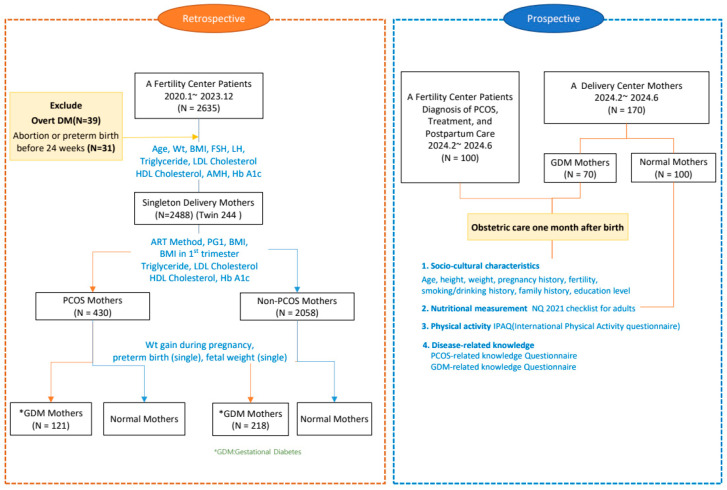
Study population and research design.

The prospective cohort study was conducted with participants who provided informed consent and completed a structured 70-item questionnaire in a counseling room at the A Hospital. The questionnaire assessed multiple domains, including general characteristics (10 questions), knowledge of PCOS (20 questions), knowledge of gestational diabetes (15 questions), nutritional status (18 questions), and physical activity (7 questions). The completion time for the questionnaire was approximately 20 min (Figure 1).

### 2.2. Study Population

The retrospective study reviewed 2635 medical records, excluding cases involving pre-existing diabetes, miscarriages occurring before 24 weeks of gestation, and preterm deliveries. Due to the nature of the electronic medical record data, we did not have control over the sample size or data collection process. For the prospective study, a descriptive cross-sectional design was employed, utilizing a face-to-face survey format. The sample size was calculated using G*Power 3.1.9.2 software (Heinrich Heine University, Düsseldorf, Germany), based on a power of 0.80, an alpha level of 0.05, and an effect size of 0.25, which determined a minimum required sample size of 89 participants. To account for potential non-responses, the recruitment target was increased by 10%, resulting in a total of 270 participants.

The inclusion criteria of the study were Korean pregnant women aged 19–45 years who had delivered following infertility treatment. Participants were categorized into three groups: those diagnosed with PCOS, those with GDM, and a normal maternal group. The exclusion criteria included individuals with pre-existing type 1 or type 2 diabetes prior to pregnancy and those unable to independently read or complete the questionnaire (Figure 1).

### 2.3. Data Collection

This study received approval from the Institutional Review Board (IRB) of the affiliated institution (#ICHA 2023-11-006). Data were collected between December 2023 and June 2024. Participants were enrolled after providing written informed consent in compliance with IRB guidelines. Regular monitoring by the research team ensured adherence to the clinical study protocol. All data were collected solely for research purposes and will be securely stored for three years following the study’s conclusion, after which they will be permanently deleted. The research team, having completed comprehensive training in research ethics, implemented rigorous measures to protect the confidentiality of participants’ personal information.

### 2.4. Study Questionnaires

In the prospective study, participants completed a self-administered questionnaire addressing several domains: general characteristics, nutrition status, physical activity status, and knowledge about GDM and PCOS. The general characteristics section included 10 questions covering age, height, weight, pregnancy history, parity, waist-to-hip ratio, smoking and drinking status, parental family history, and education level. Waist-to-hip circumference was measured by a nurse in the consultation room using a standardized measuring scale, ensuring accuracy before recording. The following sections assessed participants’ dietary habits, physical activity levels, and knowledge related to GDM and PCOS.

#### 2.4.1. Dietary Habits

The nutrition questionnaire included 18 items from the Nutrition Quotient-2021 (NQ-2021) and two additional questions on sugary beverage intake and exercise. NQ-2021 is a simplified tool assessing balance, moderation, and practice in diet through questions on food intake frequency, nutrition label use, handwashing, and risky drinking behaviors [19]. This tool is tailored to South Korean dietary patterns, with each item scored from 0 to 100. Scores are weighted (balance and moderation at 30% each, practice at 40%) to calculate a total nutrition index, offering an overall view of dietary behavior.

#### 2.4.2. Physical Activity Levels

Physical activity in the PCOS, GDM, and normal maternal groups was measured using the International Physical Activity Questionnaire—Short Form (IPAQ-SF) [20]. This questionnaire recorded the total time spent on various physical activities over the previous seven days, specifically capturing the time spent walking, engaging in moderate and vigorous physical activities, and being inactive. Participants’ responses were converted into metabolic equivalent minutes per week (MET-min/week) based on the IPAQ scoring protocol.

Physical activity levels were classified as low, moderate (over 30 min of moderate activity and/or walking on at least 5 days or 600 MET-min/week), and high (over 1500 MET-min/week on 3 days, or 3000 MET-min/week through various activities over 7 days). In a study verifying the reliability and validity of the Korean IPAQ (number of walking days, average days, active minutes, active days), the Spearman Rho coefficients ranged from 0.427 to 0.646, and the test–retest Kappa values were 0.365 to 0.620 [21].

#### 2.4.3. Knowledge Related to PCOS

This study used the knowledge-related section of a questionnaire developed in a cross-sectional study to assess women’s knowledge and perceptions of PCOS in Jordan [22]. The questionnaire was initially created with input from six women with academic expertise and six non-academics, who evaluated the face and content validity of the draft. For this study, 20 knowledge-related questions were selected after ensuring accurate translation through the forward-to-back translation process, confirming consistency in English and Korean. This method helped to maintain the integrity and cultural relevance of the questionnaire items.

#### 2.4.4. Knowledge Related to GDM

A tool to assess knowledge related to GDM was developed by the researchers based on the existing literature [23], comprising 15 questions [24]. Content validity was reviewed by two obstetricians and two nursing professors, covering essential topics: four questions on the definition, signs, and symptoms of GDM; four on GDM management; five on negative outcomes; and two on breastfeeding. Each question offered responses of “Yes”, “No”, and “I don’t know”, with 1 point awarded for correct answers and 0 points for incorrect or “I don’t know” responses. The knowledge scores were then converted into a percentage, where a higher score indicated a greater level of knowledge. The tool demonstrated good internal consistency, with a Cronbach alpha value of 0.763 [24].

### 2.5. Data Analysis

In the retrospective study analysis, maternal characteristics and obstetric outcomes between the PCOS and non-PCOS groups were compared. For continuous variables, Student’s *t*-test was used, and χ^2^ tests or Fisher’s exact test were applied for categorical data. Independent predictors of GDM and preterm birth were identified through logistic regression in multivariate analysis. Analyses were performed using SPSS version 26.0 (IBM Corp., Armonk, NY, USA), with statistical significance set at *p* < 0.05. Multicollinearity was considered to be present when the variance inflation factor was higher than 5. Normality and homogeneity of variances were verified by the Shapiro–Wilk and Levene tests, respectively.

For the prospective study analysis, descriptive statistics, including means, standard deviations, and percentiles, were used. Hierarchical multiple regression analysis assessed demographic and disease-related factors impacting metabolic disease and birth outcomes. This regression also evaluated the influence of PCOS on metabolic diseases, such as GDM and diabetes, and pregnancy outcomes.

## 3. Results

This study compared demographic and clinical characteristics between the PCOS (*n* = 430) and control (*n* = 2058) groups (Table 1). No significant difference was found in infertility age. However, BMI was significantly higher in the PCOS group, with more individuals having a BMI above 25. Hormone analysis showed an elevated LH/FSH ratio and AMH levels in the PCOS group. Additionally, triglycerides, LDL, HDL, and HbA1c levels were higher in the PCOS, along with an increased incidence of GDM. The rates of multiple pregnancies (*p* = 0.014) and preterm births were higher in the PCOS group, while fetal weight differences were not significant. Logistic regression showed a 2.94-fold increased GDM risk (95% CI: 2.22–3.90, *p* < 0.001) and a 1.55-fold increased preterm birth risk (95% CI: 1.09–2.24, *p* = 0.016) in the PCOS group after adjusting for age and BMI. The variance inflation factor was less than 5, and there was no multicollinearity between predictors.

In the prospective data analysis, participants were classified into PCOS, GDM, and normal maternal groups (Table 2). The GDM group had a slightly higher average age compared to the other groups, although height did not significantly differ across groups. The GDM group also had a higher average weight. In the PCOS group, a higher proportion of participants reported prior pregnancy and childbirth experience. The waist-to-hip ratio was highest in the GDM group. Additionally, paternal family history was more prevalent in the PCOS group, whereas maternal family history was slightly higher in the GDM group. Disease-specific knowledge was greater within each respective group, with both the PCOS and GDM groups displaying higher awarenesses about their conditions. From the analysis of physical activity and nutritional status, the PCOS group showed the highest level of physical activity, whereas the GDM group demonstrated the best nutritional status.

The further analysis of nutritional status considered demographic and pregnancy-related characteristics along with knowledge levels of PCOS and GDM (Table 3). The significant variables influencing nutritional status were age (β = 0.158, *p* = 0.009), weight (β = −0.189, *p* = 0.003), alcohol consumption (β = −0.235, *p* < 0.001), and GDM group (β = 0.165, *p* = 0.017). These results provide insight into factors affecting nutritional status among different patient groups.

Table 4 shows the results of physical activity analysis based on demographic and pregnancy-related characteristics, as well as knowledge of PCOS and GDM. The analysis showed that physical activity levels were significantly lower in smokers (B = −1245.002, β = −0.159, *p* = 0.013). In contrast, physical activity increased significantly among participants with a maternal history of PCOS (B = 1886.115, β = 0.162, *p* = 0.006). While physical activity tended to increase with higher knowledge of PCOS, this effect was not statistically significant (B = 8.203). However, higher knowledge of GDM was significantly associated with increased physical activity (B = 68.528, β = 0.147, *p* = 0.026).

## 4. Discussion

This study comprehensively analyzed the impact of PCOS and GDM on pregnancy and metabolic health using a mixed-methods approach. Retrospective analysis provided strong epidemiological evidence that PCOS is an independent risk factor for GDM, with a 2.94-fold increased risk. Additionally, the prevalence of preterm birth was significantly higher in women with PCOS, supporting the hypothesis that PCOS-related metabolic dysfunction negatively affects pregnancy outcomes.

In contrast, prospective analysis identified behavioral and lifestyle differences among women with PCOS, GDM, and pregnant women without these conditions. Women with PCOS exhibited higher levels of physical activity but poorer nutritional status compared to those with GDM. These findings highlight the need for tailored lifestyle interventions: while dietary modifications may be more beneficial for women with GDM, those with PCOS may require exercise interventions and metabolic monitoring. Collectively, these results suggest that a single approach may not be effective in managing the metabolic risks associated with PCOS and GDM.

These findings underscore the need for early screening, patient-specific education programs, and individualized interventions to mitigate the risks associated with PCOS and GDM. Future research should focus on long-term metabolic management, targeted education, and personalized management strategies to improve maternal and neonatal health outcomes.

Integrating epidemiological data from retrospective analysis with behavioral insights from prospective studies provides a more comprehensive understanding of the diverse risk factors associated with PCOS and GDM. Our retrospective findings elucidate the long-term metabolic risks and health outcomes of PCOS, while our prospective data identify behavioral differences in diet, exercise, and disease awareness that may contribute to disease progression. These combined findings support the necessity of a multifaceted, patient-specific approach to metabolic risk management through early screening, lifestyle modifications, and individualized healthcare interventions.

### 4.1. Metabolic Outcomes

Our retrospective data indicated a significantly higher BMI in the PCOS group than in the controls, with more individuals classified as overweight or obese (BMI ≥ 25 kg/m^2^). One potential explanation for this finding is that PCOS is closely associated with insulin resistance and metabolic dysfunction, leading to increased fat accumulation and difficulty in weight management [25]. This suggests a higher prevalence of excess weight in PCOS patients. This is consistent with findings that obesity is common in PCOS, supporting the need for dietary and lifestyle interventions to address overweight or obese patients and manage PCOS symptoms [25]. Studies also identify obesity, particularly abdominal, as a critical factor in PCOS development [26], which may worsen symptoms [27]. Hormone analysis showed elevated LH/FSH ratios and AMH levels in the PCOS group, indicating hormonal imbalances affecting ovarian function. High AMH levels correlate with reduced fertility in PCOS, with evidence showing lower pregnancy rates in women with elevated AMH compared to those with lower levels [28,29].

### 4.2. Negative Birth Outcomes

The retrospective analysis showed significantly higher rates of multiple pregnancies and preterm births in the PCOS group, indicating the potential adverse effects of PCOS on pregnancy outcomes. Similarly, other studies report higher pregnancy success and live birth rates in IVF for women with PCOS, but with an increased risk of miscarriage and complications [30]. PCOS-related hormonal and metabolic disturbances, such as increased androgen levels and insulin resistance, may compromise placental function, leading to a higher risk of preterm labor [31]. Logistic regression revealed a 1.55-fold higher risk of preterm birth in PCOS patients, linking PCOS to preterm delivery. A meta-analysis also found that infants of PCOS mothers were more likely to be admitted to NICUs due to low birth weight and preterm birth [26]. PCOS is a major cause of ovulatory infertility, making conception more challenging for affected women, often necessitating the use of Assisted Reproductive Technology (ART) [32]. Consequently, it is reasonable to assume that adverse pregnancy outcomes, including preterm birth, were more common in the PCOS group than in the control group. These findings underscore the need for enhanced prenatal care for PCOS patients to reduce adverse outcomes, especially preterm birth.

### 4.3. The Relationship Between PCOS and GDM

The retrospective analysis showed that HbA1c levels and GDM incidence were higher in the PCOS group, with a 2.94-fold increased GDM risk, suggesting that PCOS negatively impacts glucose metabolism in pregnancy. This highlights the need for vigilant blood sugar monitoring in PCOS patients due to their elevated diabetes risk. Supporting this, other studies indicate that women with PCOS have a two to three times higher risk of GDM compared to those without PCOS [30,31]. Pre-pregnancy factors, especially higher BMI, are significant predictors of GDM [32]. However, while some studies link PCOS to GDM, others find no significant difference in GDM risk between PCOS and non-PCOS groups [30], suggesting that BMI may play a more critical role than PCOS itself [33].

### 4.4. PCOS Knowledge and Lifestyle-Related Factors

The retrospective analysis showed significantly higher BMIs in the PCOS group, with more individuals having a BMI over 25, suggesting higher obesity rates in PCOS patients. This aligns with studies highlighting obesity’s role in PCOS, underscoring the need for lifestyle and dietary adjustments [25]. Abdominal obesity, linked to insulin resistance and hormonal imbalances, further exacerbates PCOS symptoms like irregular menstruation and hirsutism [26,27].

The higher average age in the GDM group may indicate an increased risk of developing GDM with advancing age. This aligns with existing research identifying age as an important factor in GDM risk. Additionally, the higher weights observed in the GDM group imply that being overweight or obese may significantly impact the likelihood of developing GDM, making weight management a critical preventive measure. Studies have consistently shown that women with PCOS have a considerably higher incidence of GDM during pregnancy, with rates ranging from 10.5% to 20.6%—substantially above the general population’s risk. This increased susceptibility to GDM in PCOS patients is largely attributed to insulin resistance, obesity, and a high pre-pregnancy BMI, all of which are common in PCOS. Managing these factors may help to reduce GDM risk in pregnant women with PCOS, underscoring the importance of pre-pregnancy interventions [34].

The higher rates of pregnancy and childbirth experience in the PCOS group suggest a close association between PCOS and reproductive challenges, potentially contributing to increased pregnancy and childbirth difficulties. In the GDM group, the elevated waist-to-hip ratio indicates that abdominal obesity may be a significant risk factor for gestational diabetes. Research identifies the waist-to-hip ratio as a key marker linked to diabetes and metabolic syndrome. Studies have increasingly highlighted that abdominal obesity, particularly indicators like visceral adipose tissue (VAT), is closely associated with GDM risk and may serve as a more precise predictor than BMI alone. A meta-analysis found that visceral fat depth is notably higher in individuals with GDM compared to non-GDM patients, showing a direct connection between abdominal obesity and GDM risk. Further findings demonstrate that abdominal circumference (AC) and the abdominal circumference-to-height ratio (ACHtR) are also significant predictors of GDM. Abdominal obesity is thought to induce insulin resistance in early pregnancy, which may increase the likelihood of GDM development. These insights underscore the importance of monitoring abdominal obesity as part of early GDM risk assessment and intervention strategies [35].

The higher disease knowledge in the PCOS and GDM groups suggests greater health awareness, highlighting the role of education in managing these conditions. Better nutritional status in the GDM group points to the importance of diet in diabetes management, while increased physical activity in the PCOS group underscores exercise’s benefits in managing PCOS symptoms. These findings emphasize targeted education, diet, and exercise as key strategies for managing both PCOS and GDM.

### 4.5. Study Limitation

This study has limitations. Its generalizability may be restricted as it focused on patients from a single hospital in Gyeonggi, although the age distribution closely matches national data, primarily involving patients in their 30s. The combined retrospective and prospective design, essential for long-term PCOS follow-up, limits causal interpretation due to timing variations and missing data. The prospective component’s reliance on self-reported questionnaires may affect accuracy, as participants may under-report or over-report behaviors. Additionally, some confounding factors, like lifestyle and socioeconomic status, may not have been fully controlled.

## 5. Conclusions

This study emphasizes the strong association between PCOS and GDM, demonstrating that individuals with PCOS have an increased risk of GDM and adverse pregnancy outcomes. The findings suggest that metabolic dysregulation, including insulin resistance and lipid abnormalities, is more prevalent in women with PCOS, reinforcing the need for early screening and targeted clinical interventions. Additionally, variations in dietary habits and physical activity levels among study groups highlight the importance of individualized management strategies to mitigate metabolic complications in high-risk populations.

Despite its contributions, this study has certain limitations, including sample size constraints and potential selection bias. Future research should explore the long-term metabolic and reproductive consequences of PCOS beyond pregnancy and assess the effectiveness of tailored intervention programs. Addressing these gaps will support the development of comprehensive healthcare strategies aimed at optimizing maternal and neonatal health outcomes in women with endocrine disorders.

## Figures and Tables

**Table 1:** Demographic and clinical characteristics of participants (*n* = 2635).

Variable	PCOS (*n* = 430)	Normal (*n* = 2058)	*χ*^2^ or *T*	*p*-Value
	Mean ± SD or *n* (%)			
Age in infertility	33.5 ± 3.9	33.7 ± 3.6	0.718	0.473
BMI ^1^ in infertility; kg/m^2^	27.3 ± 3.8	21.2 ± 2.4	−42.762	<0.001
BMI > 25; %	3.6	87.4	1682.904	<0.001
LH ^2^/FSH ^3^; mIU/mL	1.3 ± 1.2	1.0 ± 0.5	−9.312	<0.001
Triglyceride; mg/dL	129 ± 84	82 ± 42	−5.559	<0.001
LDL ^4^; mg/dL	133 ± 31	116 ± 26	−4.235	<0.001
HDL ^5^; mg/dL	56 ± 14	69 ± 14	6.528	<0.001
AMH ^6^; pmol/L	4.4 ± 3.6	3.8 ± 2.8	−3.497	< 0.001
Hb A1c in infertility; %	5.4 ± 0.3	5.1 ± 0.3	−7.362	<0.001
Parity (Primipara)	351 (81.6)	1732(84.2)	1.672	0.196
ART ^7^ (IVF-TET ^8^)	287(66.7)	1140(55.4)	−4.343	<0.001
Twin	56(13.0)	188(9.1)	6.079	0.014
PG1; mg/dL	137 ± 27	125 ± 24	−8.783	<0.001
Age in pregnancy	34.4 ± 3.9	34.5 ± 3.7	0.230	0.046
BMI in 1st trimester; kg/m^2^	27.4 ± 4.2	21.4 ± 2.5	−6.072	<0.001
BMI at term; kg/m^2^	30.0 ± 4.6	26.0 ± 3.1	−5.058	<0.001
GDM ^9^	121(28.1)	218(10.6)	93.051	<0.001
Hb A1c in pregnancy; %	5.2 ± 0.4	5.0 ± 0.3	−6.049	<0.001
Triglyceride in pregnancy; mg/dL	326 ± 204	272 ± 130	−2.992	0.011
LDL in pregnancy; mg/dL	144 ± 43	151 ± 40	0.685	<0.001
HDL in pregnancy; mg/dL	70 ± 17	77 ± 17	3.495	0.001
Preterm birth (single)	44/375 (16.7)	147/1873 (8.2)	4.796	0.029
Weight gain during pregnancy; kg	9.8 ± 5.7	12.3 ± 4.3	9.746	<0.001
Fetal weight (single)	3207 ± 499	3160 ± 408	−1.152	0.053

^1^ BMI = Body Mass Index. ^2^ LH = Luteinizing Hormone. ^3^ FSH = Follicle Stimulating Hormone. ^4^ LDL = low-density lipoprotein. ^5^ HDL = high-density lipoprotein. ^6^ AMH = Anti-Mullerian Hormone. ^7^ ART = Assisted Reproductive Technology. ^8^ IVF-TET = In Vitro Fertilization–Thawing Embryo Transfer. ^9^ GDM = gestational diabetes mellitus.

**Table 2:** Demographic and clinical characteristics of participants (*n* = 269).

Variable		PCOS (*n* = 99)	GDM (*n* = 70)	Normal (*n* = 100)	Total (*n* = 269)
		M ± SD or *n* (%)			
Age		33.23 (3.68)	35.67 (3.86)	33.21 (3.58)	33.86 (3.83)
Height		161.84 (4.93)	162.55 (4.89)	162.3 (5.01)	162.20 (4.94)
Weight		63.25 (13.38)	68.30 (9.62)	68.28 (8.42)	66.44 (11.02)
Pregnancy history	0	59 (59.60)	3 (4.29)	3 (3.00)	65 (24.16)
	1	29 (29.29)	51 (72.86)	64 (64.00)	144 (53.53)
	2	9 (9.09)	12 (17.14)	28 (28.00)	49 (18.22)
	3	1 (1.01)	3 (4.29)	3 (3.00)	7 (2.60)
	4	0 (0.00)	1 (1.42)	2 (2.00)	3 (1.11)
	5	1 (1.01)	0 (0.00)	0 (2.00)	1 (0.37)
Birth history	0	89 (89.90)	49 (70.00)	49 (49.00)	187 (69.52)
	1	9 (9.09)	18 (25.71)	43 (43.00)	70 (26.02)
	2	1 (1.01)	3 (4.29)	7 (7.00)	11 (4.09)
	3	0 (0.00)	0 (0.00)	1 (1.00)	1 (0.37)
Waist circumference		80.35 (12.41)	101.77 (9.91)	100.38 (12.47)	93.37 (15.44)
Hip circumference		98.27 (9.75)	104.17 (6.94)	105.56 (6.56)	102.51 (8.60)
Waist to hip ratio		0.82 (0.07)	0.96 (0.14)	0.95 (0.11)	0.90 (0.13)
Smoking (Yes)		8 (8.08)	0 (0.00)	1 (1.00)	9 (3.35)
Drinking (Yes)		38 (38.38)	12 (17.14)	28 (28.00)	78 (29.00)
Paternal family history(Yes)		37 (37.37)	38 (54.28)	36 (36.00)	111 (41.26)
Maternal family history(Yes)		41 (41.41)	40 (57.14)	27 (27.00)	108 (40.15)
	PCOS ^1^	4 (4.04)	0 (0.00)	0 (0.00)	4 (1.49)
Education	High school	13 (13.13)	8 (11.43)	9 (9.00)	30 (11.15)
	Undergraduate school	76 (76.76)	56 (80.00)	84 (84.00)	216 (80.30)
	Graduate school	10 (10.10)	6 (8.57)	7 (7.00)	23 (8.55)
PCOS ^1^ knowledge		11.25 (2.81)	11.4 (3.70)	10.71 (3.31)	11.09 (3.25)
GDM ^2^ knowledge		7.05 (2.87)	10.37 (1.75)	9.08 (3.09)	8.67 (3.02)
Nutritional status		48.75 (12.59)	58.84 (11.27)	52.07 (11.20)	52.61 (12.36)
Physical activity		1596.0(1871.71)	647.48 (824.87)	712.72 (959.57)	1020.87(1410.81)

^1^ PCOS = polycystic ovary syndrome. ^2^ GDM = gestational diabetes mellitus.

**Table 3:** Differences in nutritional status based on demographic and pregnancy-related characteristics, PCOS knowledge, and GDM knowledge.

	*B*	*S.E.*	*β*	*t*	*p*	*VIF*
Constant	2.291	24.750		0.093	0.926	
Age	0.509	0.194	0.158	2.618	0.009	1.207
Height	0.254	0.146	0.101	1.735	0.084	1.132
Weight	−0.212	0.070	−0.189	−3.026	0.003	1.293
Pregnancy history (Yes)	1.553	2.183	0.054	0.712	0.477	1.906
Birth history (Yes)	−1.955	1.658	−0.073	−1.179	0.240	1.272
Waist to hip ratio	7.916	6.686	0.081	1.184	0.238	1.551
Smoking (Yes)	−0.519	4.250	−0.008	−0.122	0.903	1.274
Drinking (Yes)	−6.395	1.592	−0.235	−4.016	<0.001	1.139
Paternal family history (Yes)	−0.846	1.460	−0.034	−0.579	0.563	1.127
Maternal family history (Yes)	−1.002	1.487	−0.040	−0.674	0.501	1.160
Maternal PCOS history (Yes)	3.098	5.812	0.030	0.533	0.595	1.080
Education (ref. = graduate school)						
High school	−1.706	3.234	−0.044	−0.527	0.598	2.261
Undergraduate school	−1.668	2.510	−0.054	−0.665	0.507	2.175
Group (ref = normal)						
PCOS	−2.794	2.340	−0.109	−1.194	0.234	2.779
GDM	4.630	1.919	0.165	2.412	0.017	1.547
PCOS knowledge	0.276	0.218	0.073	1.264	0.207	1.094
GDM knowledge	−0.097	0.260	−0.024	−0.373	0.709	1.345
*R*^2^/*R*^2^*adj./D-W*	0.244/0.193/1.859					
*F(p)*	4.764 (<0.001)					

**Table 4:** Differences in physical activity according to demographic and pregnancy-related characteristics, PCOS knowledge, and GDM knowledge.

	*B*	*S.E.*	*β*	*t*	*p*	*VIF*
Constant	−608.923	2905.247		−0.210	0.834	
Age	−16.249	22.829	−0.044	−0.712	0.477	1.207
Height	1.828	17.154	0.006	0.107	0.915	1.132
Weight	4.956	8.217	0.039	0.603	0.547	1.293
Pregnancy history (Yes)	−341.334	256.252	−0.104	−1.332	0.184	1.906
Birth history (Yes)	73.959	194.673	0.024	0.380	0.704	1.272
Waist to hip ratio	852.999	784.855	0.076	1.087	0.278	1.551
Smoking (Yes)	−1245.002	498.836	−0.159	−2.496	0.013	1.274
Drinking (Yes)	−219.048	186.927	−0.071	−1.172	0.242	1.139
Paternal family history (Yes)	−138.790	171.335	−0.049	−0.810	0.419	1.127
Maternal family history (Yes)	−211.482	174.568	−0.074	−1.211	0.227	1.160
Maternal PCOS history (Yes)	1886.115	682.259	0.162	2.765	0.006	1.080
Education (ref. = graduate school)						2.261
High school	555.789	379.615	0.124	1.464	0.144	2.175
Undergraduate school	153.868	294.641	0.043	0.522	0.602	2.779
Group (ref = normal)						1.547
PCOS	1051.754	274.681	0.360	3.829	<0.001	1.094
GDM	−73.957	225.277	−0.023	−0.328	0.743	1.345
PCOS knowledge	8.203	25.640	0.019	0.320	0.749	1.207
GDM knowledge	68.528	30.578	0.147	2.241	0.026	1.132
*R*^2^/*R*^2^*adj./D-W*	0.201/0.147/2.013					
*F(p)*	3.707 (<0.001)					

## Data Availability

The data supporting the findings of this study are available from the corresponding author upon reasonable request.
